# Nodular Fasciitis of the Orbit: A Case Report and Brief Review of the Literature

**DOI:** 10.1155/2011/235956

**Published:** 2011-10-19

**Authors:** John E. Riffle, Andrea H. Prosser, Jeffery R. Lee, Julie J. Lynn

**Affiliations:** ^1^Ophthalmology Department, Charlie Norwood VA Medical Center, 1 Freedom Way, Augusta, GA 30904, USA; ^2^Ophthalmology Department, Georgia Health Sciences University, 1120 15th Street, Augusta, GA 30912, USA; ^3^Pathology and Laboratory Medicine Department, Charlie Norwood VA Medical Center, 1 Freedom Way, Augusta, GA 30904, USA; ^4^Ophthalmology Department, Eye Physicians and Surgeons of Hattiesburg, 415 South 28th Avenue, Hattiesburg, MS 39401, USA

## Abstract

Nodular fasciitis is a benign, reactive, fibroblastic proliferation in which nodules most commonly develop in the subcutaneous or superficial fascia of the extremities. The occurrence of these growths in the orbital region is relatively rare. Our intent is to report another orbital case of this benign fibroproliferative tumor and to provide a brief review of the pertinent medical literature. The salient pathologic features of nodular fasciitis are summarized. The potential for the misdiagnosis of these benign mesenchymal tumors as a malignant sarcomatous neoplasm is discussed. It is important for ophthalmologists to be aware of this pathologic entity and its pseudosarcomatous appearance.

## 1. Introduction

Nodular fasciitis is a benign, reactive, fibroblastic proliferation in which nodules most commonly develop in the subcutaneous or superficial fascia of the extremities. Font and Zimmerman first described nodular fasciitis occurring in the periorbital area. They presented 10 cases located on the eye or ocular adnexa [1]. In *Shields and Shields* textbook on eyelid, conjunctival and orbital tumors orbital nodular fasciitis accounts for only 2 cases out of their series of 1,264 orbital lesions [2]. The potential for the misdiagnosis of this benign growth as a malignant neoplasm is under recognized. The intent of this paper is to report an additional case of this relatively rare benign orbital fibroproliferative neoplasm.

## 2. Case Report

**Figure 1:**
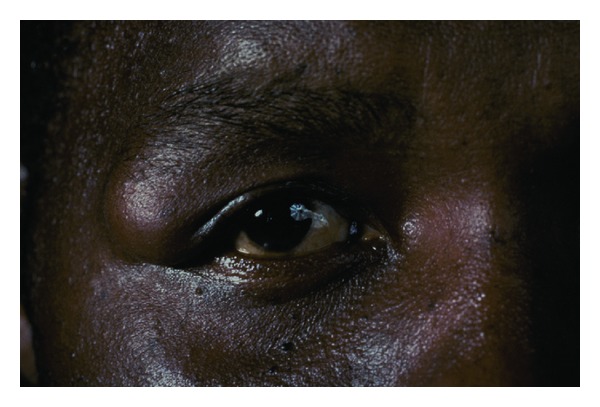
Mass at temporal orbital rim.

**Figure 2:**
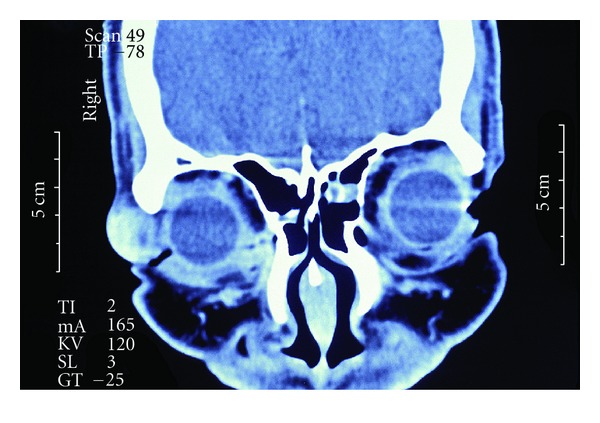
CT scan of right orbital rim mass.

**Figure 3:**
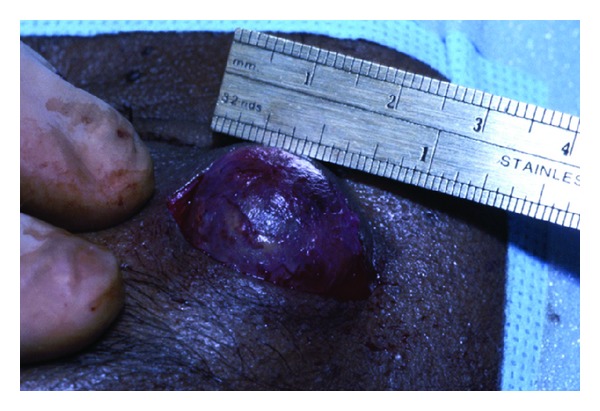
Partially dissected mass.

A 45-year-old African American male presented with a complaint of a gradually enlarging mass at the lateral aspect of the right upper eyelid. This growth was initially noted three months prior to presentation and was not associated with pain. There was no history of trauma to the region. On examination, the uncorrected acuity was 20/25 in each eye. A firm nontender, partially mobile mass was palpable over the temporal aspect of the superior orbital rim of the right eye (Figure 1). The growth measured 2.5 cm in diameter. There were no other abnormal findings on the general eye examination. A CT scan disclosed a well-demarcated mass with no infiltration into surrounding tissues (Figure 2). A few weeks later, the patient was taken to surgery and the growth was removed under local anesthesia. A 3 cm incision was made directly over the mass and blunt dissection was used to free the mass from the surrounding tissue (Figure 3). The growth was quite adherent to the deep tissue overlying the orbital rim and appeared to be encapsulated. 

**Figure 4:**
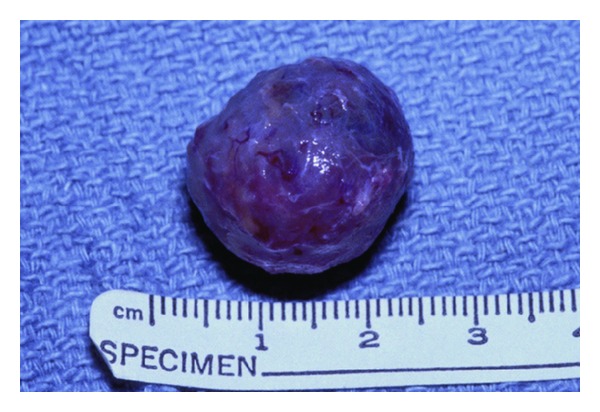
Gross operative specimen.

**Figure 5:**
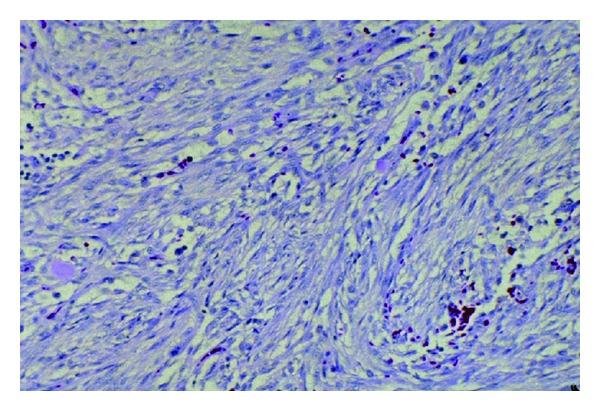
Histologic specimen revealing tapered spindle cells.

**Figure 6:**
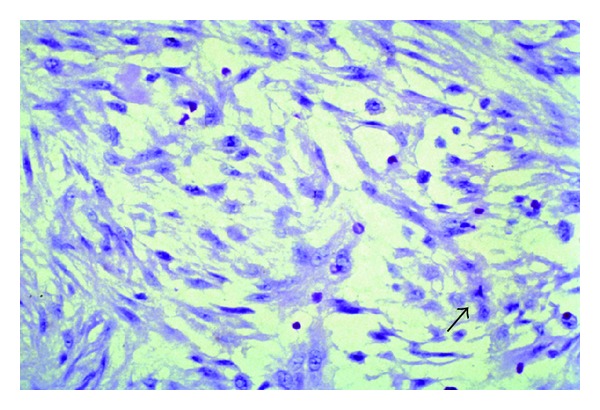
Arrow points to mitosis on high magnification.

The specimen was submitted to pathology. It was noted to be a 2 cm well-circumscribed mass (Figure 4). On histological examination, the tumor was described as being surrounded by compressed bands of collagen, but a true capsule was not present. It consisted of bundles of finely tapered spindle cells that resembled fibroblasts found in tissue culture or granulation tissue (Figure 5). There was abundant myxoid stoma with scattered lymphocytes and extravasated red blood cells. Occasional mitoses were identified (Figure 6). In order to exclude other spindled neoplasms, an immunohistochemical analysis was performed utilizing antibodies against S-100, epithelial membrane antigen, cytokeratin, muscle actin, and neurofilament antigen. These studies were all were negative. 

The patient did well postoperatively. The wound healed without complication and there has been no recurrence of the growth.

## 3. Discussion

Nodular fasciitis was first discovered by Konwaler et al. as a distinct clinical entity [3]. Nodular fasciitis occurs in all age groups with an equal incidence in both male and female. The preponderance of these tumors arise from the subcutaneous fascia of the truck and upper extremities. Nodular fasciitis is rarely observed on the head or neck of adults; however, these locations are common in infants and children [4, 5]. In children it tends to occur in the anterior periorbital tissues, but it has also been reported to occur in the orbit where it can simulate a dermoid cyst [6]. Additional cases have been reported originating in the tenons capsule [7, 8] and episclera [9]. Also, an epibulbar nodular fasciitis has been reported in a patient with floppy eyelids who described frequent and vigorous eye rubbing [10]. 

Nodular fasciitis characteristically has a rapid growth which can clinically simulate a rhabdomyosarcoma [2]. Additionally its pleomorphic spindle cell pattern and mitotic activity can histologically be confused with a fibrosarcoma or other soft tissue tumor [5, 11]. This high index of suspicion for a malignancy can lead to unnecessary aggressive treatment [12]. Imaging studies show no distinctive features to separate nodular fasciitis from other solid masses. While nodular fasciitis would seem to be suitable for fine needle aspiration diagnosis there are few reports of nodular fasciitis being diagnosed cytologically [13]. 

Nodular fasciitis is typically located anteriorly in the periorbital area and is well circumscribed. The majority of these lesions are uncapsulated growths measuring less than 3 cm in diameter. Complete tissue sparing surgical excision is recommended when at all feasible. Local reoccurrence is reported as rare following complete excision [11]. 

While the histopathologic diagnosis of nodular fasciitis can be problematic most experienced pathologists can make the diagnosis based on the light microscopic features. Classically there is the proliferation of spindle cell fibroblasts which are frequently arranged in parallel bundles extending in all directions resembling cells in tissue culture. While mitoses may be numerous the spindle cell nuclei are never hyperchromatic and atypical mitoses are virtually never seen.

In summary, we present a case report of a rapidly developing fibroblastic proliferation located at the lateral superior orbital margin in a 45-year-old male. While nodular fasciitis accounts for considerably less than 1 percent of all orbital lesions, it is important that ophthalmologists be aware of this pathologic entity and its potential for misdiagnosis as a malignant neoplasm.
